# Functional brain networks underlying the interaction between central and peripheral processes involved in Chinese handwriting in children and adults

**DOI:** 10.1002/hbm.26055

**Published:** 2022-08-25

**Authors:** Junjun Li, Ying Liu, Yi Wang, Nizhuan Wang, Yuzhu Ji, Tongqi Wei, Hong‐Yan Bi, Yang Yang

**Affiliations:** ^1^ CAS Key Laboratory of Behavioral Science, Center for Brain Science and Learning Difficulties Institute of Psychology, Chinese Academy of Sciences Beijing China; ^2^ Department of Psychology University of Chinese Academy of Sciences Beijing China; ^3^ School of Medical Humanities Capital Medical University Beijing China; ^4^ School of Mechanical and Materials Engineering North China University of Technology Beijing China; ^5^ School of Biomedical Engineering ShanghaiTech University Shanghai China; ^6^ Artificial Intelligence and Neuro‐Informatics Engineering (ARINE) Laboratory School of Computer Engineering, Jiangsu Ocean University Lianyungang China; ^7^ Department of Psychology, College of Education Zhejiang University of Technology Hangzhou China; ^8^ Pan Shuh Library Institute of Psychology, Chinese Academy of Sciences Beijing China

**Keywords:** brain network, central processing, handwriting, interaction, peripheral processing

## Abstract

The neural mechanisms that support handwriting, an important mode of human communication, are thought to be controlled by a central process (responsible for spelling) and a peripheral process (responsible for motor output). However, the relationship between central and peripheral processes has been debated. Using functional magnetic resonance imaging, this study examined the neural mechanisms underlying this relationship in Chinese handwriting in 36 children (mean age = 10.40 years) and 56 adults (mean age = 22.36 years) by manipulating character frequency (a central variable). Brain network analysis showed that character frequency reconfigured functional brain networks known to underlie motor processes, including the somatomotor and cerebellar network, in both children and adults, indicating that central processing cascades into peripheral processing. Furthermore, the network analysis characterized the interaction profiles between motor networks and linguistic‐cognitive networks, fully mapping the neural architecture that supports the interaction of central and peripheral processes involved in handwriting. Taken together, these results reveal the neural interface underlying the interaction between central and peripheral processes involved in handwriting in a logographic writing system, advancing our understanding of the neural basis of handwriting.

## Graphical abstract

Using functional magnetic resonance imaging, this study offers novel neural evidence for the interaction between central and peripheral processes in handwriting from the perspective of functional brain networks. The manipulation of a central variable induced reconfigurations of the somatomotor network and the cerebellar network, networks known to serve motor processes, in both children and adults. Furthermore, children and adults exhibited differences in internetwork connectivity between the somatomotor network and the auditory and visual networks, suggesting the development of the brain systems supporting the interaction.

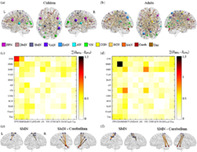

## 1 INTRODUCTION

Handwriting is an important form of language production that requires complex linguistic, cognitive and perceptual‐motor operations. Broadly, handwriting is divided into two streams of processing, characterized as “central” and “peripheral”. The central component refers to linguistic‐cognitive processing for the retrieval of appropriate words and correct orthographic forms via orthographic long‐term memory (the lexical route) or phoneme‐to‐grapheme conversion (the sublexical route); the peripheral component refers to motor processing including allograph selection, motor planning and execution of specific motor programs (Ellis, 1982).

In this conceptualization, the flow of information between central and peripheral processing streams is fundamental for understanding the mechanisms of handwriting. However, the literature on this topic is limited and conflicted. Evidence from neuropsychological studies has suggested that central and peripheral processes are dissociable: patients with central dysgraphia who have impaired spelling processing often exhibit writing difficulty regardless of motor output modality (handwriting, oral spelling, or typing), whereas patients with peripheral dysgraphia are characterized by specific difficulties with motor processing but exhibit preserved oral spelling ability (Baxter & Warrington, 1986). Behavioral and neuroimaging studies provided support for this alternative independence hypothesis. For example, a prior behavioral study showed that phonological and orthographic similarity did not influence the motor duration of handwriting, suggesting that lexical processing is independent of motor responses (Damian & Stadthagen‐Gonzalez, 2009). Furthermore, functional neuroimaging studies have demonstrated that central and peripheral processes have distinct brain regions. For instance, the left inferior frontal gyrus and fusiform gyrus assist with the central component, while the left posterior middle frontal gyrus (Exner's area), inferior/superior parietal lobule, and cerebellum serve the peripheral component (Planton et al., 2013; Purcell et al., 2011).

Conversely, multiple behavioral studies have found that manipulating linguistic variables modulates motor responses in handwriting, implying that the central and peripheral processes interact (Delattre et al., 2006; Kandel et al., 2013; Kandel & Perret, 2015; Planton et al., 2013; Zhang & Feng, 2017). For example, word frequency (Delattre et al., 2006; Kandel & Perret, 2015) and lexicality (the lexical variable) (Roux et al., 2013; Zhang & Feng, 2017) impact motor duration and motor fluency. Moreover, some studies have demonstrated that orthographic regularity (the sublexical variable) influences motor processing in writing‐to‐dictation (Delattre et al., 2006) and copying tasks (Lambert et al., 2011). Furthermore, by analyzing the motor duration of each letter, Roux et al. (2013) reported that the lexical and sublexical variables affected motor processes in different manners. The influence of lexicality occurs at the initial position of the word, whereas orthographic regularity impacts motor duration throughout the whole word.

Beyond the behavioral evidence, recent interest has been directed toward the neural mechanisms of the interaction between central and peripheral processes involved in handwriting. For example, a functional magnetic resonance imaging (fMRI) study reported that brain activation in the motor regions (e.g., the left superior parietal lobule, left superior frontal gyrus, and right cerebellum) of adults is mediated by manipulating orthographic regularity (Palmis et al., 2019). In the same vein, another study showed the effects of word frequency in brain activation in motor regions, including the left premotor area and superior parietal lobule (Yang et al., 2018). More recently, a transcranial magnetic stimulation study showed that stimulating Exner's area, a brain locus for peripheral process, induced an increase in writing speed for inconsistent words, but not for consistent words, during a writing‐to‐dictation task. This result implies that brain activity in motor regions is sensitive to the sublexical features of words (Planton et al., 2019). Although the aforementioned studies have provided primary neural evidence for the interaction between central and peripheral processes, how these related regions interact with each other in the interaction remains far from clear.

On the other hand, although evidence of the interaction between central and peripheral components is substantial in adults, it remains largely unknown when and how this interaction is constructed during the development of handwriting. A few behavioral studies have addressed this question in children. Using a copying task, Kandel and Perret (2015) examined the interaction between central and peripheral processes in French children from ages 8 to 10 by manipulating orthographic regularity and word frequency. They found that this interaction was present by age 8 and became adultlike at ages 9 and 10. Interestingly, lexical processing influences motor processing at the beginning of words at age 8, but at both the beginning and end of words at ages 9 and 10, implying an increase in the interaction in this time period. However, another study that examined word frequency in a copying task and a spelling‐to‐dictation task reported a decreasing trend in the interaction between central and peripheral processes in Spanish children from grades 2 to 6. Specifically, the interaction was significant at grade 2, marginally significant at grade 4 and disappeared at grade 6. Such a decrease in the interaction of central and peripheral processes is thought to reflect the progressive independence of the two components due to motor automatization (Afonso et al., 2018). Regardless, the above findings suggest that the pattern and extent of the interaction between central and peripheral processes in handwriting vary with the development of handwriting skills in alphabetic languages.

Chinese is a logographic writing system that is considerably different from alphabetic languages. Unlike the linear structure of alphabetic letters, Chinese characters (the basic written unit) have a square configuration. In addition, regular correspondence between orthography and phonology is lacking in Chinese. Moreover, Chinese characters include a large number of homophones with a single syllable shared by many characters, forming an extremely deep orthography. These characteristics are likely to influence orthographic access and motor complexity in Chinese handwriting. Correspondingly, the brain system that supports Chinese handwriting is found to exhibit specificity. For example, the left middle frontal gyrus exhibits greater activation in Chinese handwriting than in alphabetic languages (Cao & Perfetti, 2016). The right fusiform gyrus is specifically engaged in Chinese handwriting (Yang et al., 2018; Yang et al., 2019). However, the neural correlates of the interaction between central and peripheral processes in Chinese handwriting have rarely been investigated.

Given this background, the present study sought to examine the neural basis of the interaction between central and peripheral processes in Chinese handwriting in both children and adults. We applied a similar experimental logic as that used by previous behavioral (Kandel & Perret, 2015) and fMRI (Palmis et al., 2019) studies. Namely, if the manipulation of central variables induced brain responses for motor processes, we could conclude that central processing cascades into motor execution involved in handwriting. In the present study, character frequency was used as the central variable for the following reasons. First, the word frequency effect has been widely shown to tease apart central and peripheral processes in handwriting, both in children and adults (Afonso et al., 2018; Kandel & Perret, 2015). Second, previous studies have demonstrated that the word frequency effect occurs at the lexical‐semantic level or orthographic level (Bonin et al., 2016; Qu et al., 2016; Wang & Zhang, 2021), both of which are constrained within the central component. Third, the impact of word frequency on motor execution is consistent at all ages (Kandel & Perret, 2015), and thus, it is suitable for use in examining neural mechanisms across age groups. Furthermore, the motor complexity of characters, as evaluated by the number of strokes, was matched between the high‐frequency and low‐frequency conditions.

We used a network‐based analysis approach to measure brain responses to the manipulation of the central variable, as Chinese handwriting is a complex linguistic‐motor task that likely involves a large‐scale brain network. Functional network analysis is a powerful tool for characterizing the macroscale synchronization of brain activity and has been widely used for establishing the relation between the topology of brain organization and cognitive processes (Cole et al., 2013; Finc et al., 2017). We hypothesized that the effect of character frequency would be observed in functional brain networks involved in orthographic access (e.g., the visual network [VN]) and motor processing (e.g., the somatomotor network [SMN]) in both children and adults. Additionally, the bilateral hemispheric regions were expected to be involved in the character frequency effect during Chinese handwriting, based on the findings of brain activation analyses, which indicated that the right motor and visual regions were uniquely involved in Chinese handwriting (Cao & Perfetti, 2016; Yang et al., 2019; Yang et al., 2020). Finally, as behavioral studies have demonstrated that the pattern and extent of the interaction vary with age (Afonso et al., 2018; Kandel & Perret, 2015), we expected to observe differences in the connectivity patterns of networks involved in the interaction between children and adults.

## 2 MATERIALS AND METHODS

### 2.1 Participants

Here, 36 children (15 males; 9.15–11.11 years old) and 56 adults (28 males; 19–28 years old) were recruited to participate in the study. All the participants were native Chinese speakers and were right‐handed, as assessed by a handedness inventory (Snyder & Harris, 1993). All participants had normal hearing and normal or corrected‐to‐normal vision, and did not suffer from any neurological diseases or psychiatric disorders. The study was approved by the ethics committee of the Institute of Psychology, Chinese Academy of Sciences. Informed consent was obtained from each adult participant and the guardian of each child participant prior to the experiment. Detailed participant information is presented in Table 1.

**TABLE 1:** Demographic information and performance on behavioral tests, presented as the mean and standard deviation

		Children (*n* = 36)	Adults (*n* = 56)
Age (years)		10.40 (0.54)	22.36 (2.32)
Sex (male/female)		15/21	28/28
*Handwriting skill*			
Writing quality		25.55 (6.08)	25.19 (6.25)
Writing speed (strokes/s)		2.67 (0.59)	3.41 (0.57)
*Visuomotor skill*			
Visuomotor integration quality^a^		50.84 (4.09)	48.71 (7.90)
*Linguistic skills*			
Reading fluency (characters/s)		1.68 (0.33)	4.68 (0.80)
Phonological awareness		28.11 (2.04)	29.18 (1.21)
*Orthographic awareness* ^b^			
Real characters	ACC	0.91 (0.06)	0.98 (0.02)
	RT (ms)	802 (134)	547 (78)
Pseudocharacters	ACC	0.70 (0.19)	0.54 (0.22)
	RT	992 (213)	881 (239)
Noncharacters	ACC	0.92 (0.09)	0.96 (0.05)
	RT	802 (119)	603 (79)

Abbreviations: ACC, accuracy; ms, milliseconds; RT, reaction time.

^a^ The data of one child participant were missing.

^b^ The data of two children and four adults were missing.

### 2.2 Stimuli and task procedure

Both child and adult participants performed a delayed copy task during an fMRI scan. To minimize potential head motion artifacts in the fMRI data, they were instructed to write while minimizing movements of their upper arm and forearm. To approximate real handwriting, immediate visual feedback (“ink”) was provided on the display screen during responses. Furthermore, participants were required to write in a stroke‐by‐stroke manner to ensure that the stroke served as a motor unit.

A block design was employed for the fMRI scan in both children and adults. However, there were slight differences in the experimental design of the copying task between children and adults. For children, the stimuli included 32 characters, of which half were of “high frequency” characters (HFCs; mean frequency = 1375 times per million) and half were of “low frequency” characters (LFCs; mean frequency = 50 times per million) according to the Modern Chinese Frequency Dictionary (1986). The mean number (standard deviation, SD) of strokes was 5.56 (0.63) for HFCs and 5.35 (0.72) for LFCs. Each participant underwent two fMRI “runs”. A drawing symbols condition and a direct copying condition were included as part of a larger study, but were not analyzed in the present study, because they were not directly relevant to the effects of character frequency. Each run consisted of two blocks of copying HFCs, two blocks of copying LFCs, two blocks of drawing symbols and two blocks of direct copying, presented in pseudorandom order. Each block started with visual presentation of the instructions for 2 s, followed by four trials. In each trial, a fixation cross (+) was presented in the center of the screen for 0.5 s, followed by the presentation of a character stimulus for 1.2 s. Next, a blank screen was displayed during a delay period of 0.5 s; afterward, the cursor appeared in the central of the screen to allow participants to copy/draw during the response period of 5.3 s. The task blocks in each run alternated with eight blocks of an additional rest condition that lasted for 12 s.

For adults, 30 characters were selected as stimuli, consisting of 15 HFCs and 15 LFCs. The mean number (SD) of strokes was 6.00 (1.46) for HFCs and 6.00 (1.41) for LFCs. Each participant underwent an fMRI “run” consisting of three blocks of copying HFCs (mean frequency = 1035 times per million), three blocks of copying LFCs (mean frequency = 0.5 times per million) and three blocks of drawing symbols in a pseudorandom order. Each block included five trials. In each trial, a fixation cross (+) was presented in the center of the screen for 0.3 s, followed by presentation of a character stimulus for 1 s and then a response period of 4.7 s. Three blocks of the central fixation point, each with a 12 s duration, were interspersed among the copying and drawing blocks as a “rest” condition in each run.

An MRI‐compatible tablet system was used for recording behavioral data; this tablet system included a touch‐sensitive surface, a force‐sensitive stylus and an adjustable support frame (Tam et al., 2011). The support frame was carefully adjusted for each participant to ensure that handwriting could be comfortably performed throughout the imaging session and to enable tablet interaction with the forearm or wrist resting on the support such that there was no fatigue from handwriting against gravity.

### 2.3 Imaging acquisition

Imaging was performed with a 3 T MRI system (MAGNETOM Prisma^fit^, Siemens, Erlangen, Germany) at the Beijing MRI Center for Brain Research of the Chinese Academy of Sciences. Functional MRI time series data with blood oxygenation level‐dependent (BOLD) contrast were acquired using a two‐dimensional, T2*‐weighted, gradient‐echo echo planar imaging sequence (Moeller et al., 2010) (repetition time [TR] = 1000 ms, echo time [TE] = 30 ms, slice thickness = 2.2 mm, in‐plane resolution = 2.2 mm × 2.2 mm, flip angle = 45°). A total of 64 axial slices were collected. High spatial resolution anatomical images were acquired using a three‐dimensional T1‐weighted, magnetization‐prepared rapid acquisition gradient echo sequence (TR = 2200 ms, TE = 3.49 ms for children and 2.08 ms for adults, slice thickness = 1 mm, in‐plane resolution = 1.0 mm × 1.0 mm and flip angle = 8°).

### 2.4 Behavioral tests

After the fMRI, participants performed a series of behavioral tests to measure handwriting, visuomotor integration (VMI), and linguistic skills.

#### 2.4.1 Handwriting motor skill tests

A pen‐and‐paper copying test was administered to assess motor quality and speed of handwriting. Child participants were required to copy 48 Chinese characters that varied in frequency and visual complexity. Adult participants were required to copy 40 Chinese characters that varied in frequency. Writing quality was evaluated by two independent examiners using a 7‐point Likert scale (1 = very bad and 7 = very good) based on six dimensions, including stroke form, slant, organization of radicals, neatness, average size and overall appearance (Gimenez et al., 2014; Yang et al., 2020). The sum of subscores across all six dimensions was used as the final score. The interrater reliability of the assessment was high (children: intraclass correlation coefficient [ICC] = 0.92; adults: ICC = 0.91). Writing speed was calculated by dividing the number of strokes by the writing time.

#### 2.4.2 Visuomotor skill tests

A VMI test was administered to assess participants' skills in VMI. In this test, participants were asked to copy 12 geometric symbols that varied in visual complexity as accurately as possible. The stimuli were selected from the Beery‐Buktenica Developmental Test of VMI (Beery & Beery, 2010). Two independent evaluators assessed the similarity between the samples and participants' responses using a 7‐point Likert scale. The sum of the rating score for each symbol was calculated, and the mean of the two evaluators' total scores was taken as the index of VMI skill. The interrater reliability of the assessment was high in children (ICC = 0.96) and adults (ICC = 0.87).

#### 2.4.3 Linguistic skill tests

Participants' linguistic skills were evaluated in terms of phonological awareness, orthographic awareness, and reading fluency. An oddity test was used to assess phonological awareness for both children and adults. In this test, the participants were required to listen carefully to three syllables, one of which did not share the initial sound, final sound, or tone with the other two syllables (10 items for each type). The participants were asked to orally identify the odd syllable. The score was the total number of items answered correctly (maximum score = 30). The orthographic awareness test required participants to judge whether the visually presented characters on the screen were real Chinese characters. The test materials included 40 real Chinese characters, 20 pseudocharacters, and 20 noncharacters. Participants' accuracy and reaction time in response to the real characters, pseudocharacters, and noncharacters were used as scores of orthographic awareness. Finally, reading fluency was assessed. The material slightly differed between children and adults. For children, the test consisted of 160 Chinese characters of high and medium frequency, and participants were asked to read these characters aloud as fast and accurately as possible during 1 min. For adults, reading fluency was evaluated by reading 40 two‐character words aloud as quickly and accurately as possible. Performance was scored as the number of correctly named characters per second.

## 3 DATA ANALYSIS

### 3.1 Behavioral data

Descriptive results of the out‐of‐scanner behavioral tests were calculated. However, statistical comparisons for some of the tests were not conducted due to the differences in material.

For the in‐scanner behavioral performance, paired sample *t* tests were conducted to examine difference in writing duration and latency between copying HFCs and LFCs. Writing latency was defined by the time between the appearance of the response screen and the start of the response (first contact with the tablet), while the writing duration was defined from the start of the response to the end of the written response.

## 4 fMRI DATA ANALYSIS AND STATISTICS

### 4.1 Preprocessing

Image preprocessing and statistical analyses were conducted using SPM12 freeware (http://www.fil.ion.ucl.ac.uk/spm/, Wellcome Department of Cognitive Neurology, University College London, London). The fMRI time series data for each participant were first corrected for head motion; the corrected images were coregistered to the associated anatomical imaging data. The anatomical images were then transformed into Montreal Neurological Institute (MNI) stereotactic space, and the resulting transformation parameters were applied to yield fMRI time series data normalized in MNI space with cubic voxels, at a spatial resolution of 2 mm × 2 mm × 2 mm. These images were then spatially smoothed using an isotropic Gaussian kernel template with 6‐mm full‐width at half‐maximum. The head motions of all adult participants were less than 3 mm translation and 3° rotation for all scans. Six child participants were excluded from the data analysis because of excessive head motion (>3 mm translation or >3° rotation) throughout the scanning period in both runs. In addition, seven child participants were excluded due to poor quality of T1‐weighted images.

### 4.2 Functional network analysis

#### 4.2.1 Creation of functional connectivity matrices

Functional connectivity (FC) matrices were created using the CONN FC toolbox (Whitfield‐Gabrieli & Nieto‐Castanon, 2012). A validated parcellation template consisting of 264 functional regions in spheres with a 10‐mm diameter was used to define regions of interest (Power et al., 2011). BOLD time series signals corresponding to the HFC and LFC conditions were separately extracted and concatenated over blocks. Nuisance BOLD signal fluctuations from the cerebrospinal fluid and white matter were estimated and removed using the anatomical component correction (CompCor) strategy (Behzadi et al., 2007). Additionally, head motion (six motion parameters and six first‐order temporal derivatives) as well as the main effect of task were regressed out. The data were high‐pass filtered at 0.008 Hz to preserve task‐relevant high‐frequency signals. Next, we computed Pearson's correlation coefficients between each pair of regional time series signals and transformed them into Fisher's z scores. Following this procedure, undirected and weighted 264 × 264 FC matrices were constructed for each condition and for each participant.

### 4.3 Network‐based statistics

The network‐based statistic (NBS) approach was used to identify differences in functional networks between the HFC and LFC conditions (Zalesky et al., 2010; Zalesky et al., 2012). Specifically, we first performed a one‐sample *t* test (false discovery rate corrected *p* < .05) using the GRETNA toolbox (http://www.nitrc.org/projects/gretna/) (Wang et al., 2015), and found significant nonzero connections in FC matrices for the HFC and LFC conditions. Then, the FCs shared by the HFCs and LFCs conditions were retained by applying a mask derived by performing a union operation of the significant nonzero edges of the two conditions. Finally, the paired sample *t‐*test comparison was performed on FC matrices between the HFC and LFC conditions for each participant group. To account for the potential confounding effect of behavioral performance, the writing duration in each condition was included as a covariate. The edges that survived the threshold of *p* < .01 were retained, forming subnetworks for subsequent statistical comparisons. Nonparametric permutation tests with 5000 permutations were performed to estimate the significance of each subnetwork based on their intensity (the sum of test statistic values across all connections). The *p* value for a subnetwork of a given size was calculated as the proportion of permutations for which the largest component was the same size or greater. Additionally, the nodes of the identified network were assigned to 10 well‐established large‐scale networks as defined previously (Cole et al., 2013; Power et al., 2011). Finally, subnetworks with a familywise error rate‐corrected *p* < .05 were retained. The results were visualized using BrainNet Viewer (Xia et al., 2013).

Furthermore, hubs of the functional network were defined as nodes with a node strength 1.5 SD greater than the mean strength of all nodes in the network (Liu et al., 2018). Node strength is analogous to node degree, which is defined as the sum of edge weights (i.e., Fisher's z scores) attached to a node, in weighted networks (Fornito et al., 2016).

### 4.4 Brain–behavior correlation analysis

To further confirm the role of brain networks in the interaction between central and peripheral processes involved in handwriting, we examined the correlations between network connectivity strength and behavioral performance on handwriting motor and linguistic skills in children and adults. We paid particular attention to motor‐related functional networks because the responses of motor networks were the key evidence of an interaction between central and peripheral processes. We applied multiple regression analysis, in which FC strength was included as the dependent variable and the behavioral indicators, age and sex were included as independent variables. A backward elimination procedure was used to exclude redundant independent variables.

### 4.5 Validation analysis

To evaluate the robustness of our results, we performed several validation procedures. First, the network analysis was repeated by alternative procedures: (1) using a more stringent threshold of *p* < .005 to define edges in the paired sample *t* test; and (2) testing the significance of each subnetwork on the basis of another estimation method, NBS extent (the total number of connections within a component), to compare brain networks between the HFC and LFC conditions.

Second, we assessed whether and to what extent head motion influenced the results. Head motion was quantified by framewise displacement (FD), a composite score that represents instantaneous head motion (Power et al., 2012). First, a paired sample *t* test was applied to examine the differences in mean FD between the HFC and LFC conditions in children and adults. Then, a regression analysis was conducted to evaluate the correlations between mean FD and the connectivity strength of the functional network in relation to the effect of character frequency, after controlling for age and sex. Finally, we repeated the NBS analysis by including the mean FD as a covariate.

## 5 RESULTS

### 5.1 Behavioral results

The results of the out‐scanner behavioral tests are presented in Table 1. Due to technical reasons, some participants' data were missing for the visuomotor skill test (one child) and the orthographic awareness test (two children and four adults).

**FIGURE 1:**
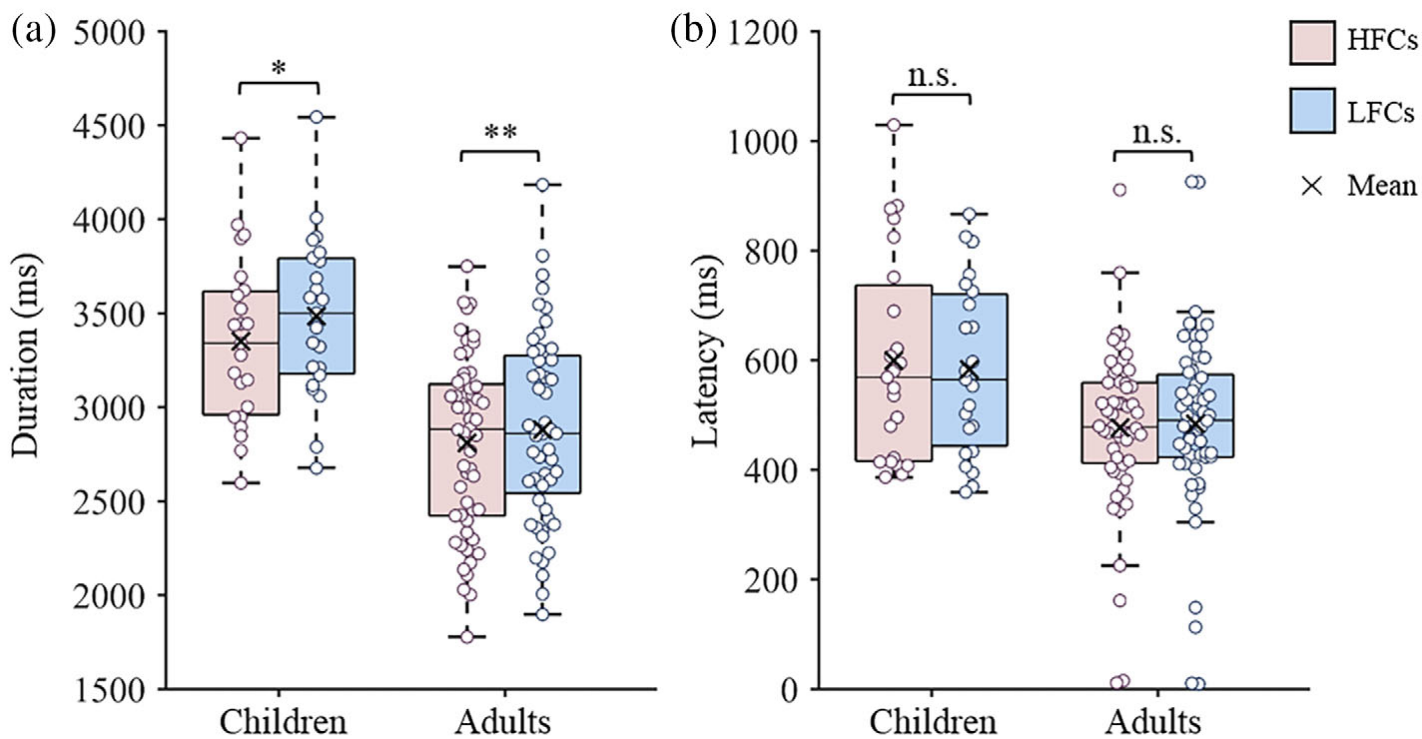
Descriptive statistics and analysis of in‐scanner behavioral performance. The writing duration (a) and latency (b) of children and adults while copying Chinese characters. HFCs, high‐frequency characters; LFCs, low‐frequency characters. n.s., not significant. *p < .05, **p < .01

Figure 1 presents the results of the in‐scanner behavioral performance; specifically, the writing duration results in children and adults are presented in Figure 1(a). The results of children were based on 23 child participants (mean age = 10.37, 15 females) after screening for fMRI data quality. Paired sample *t* tests showed that the writing duration of HFCs was significantly lower than that of LFCs (*t*(22) = −2.70, *p* = .013). For adults, paired sample *t* tests showed that the writing duration of HFCs was significantly lower than that of LFCs (*t*(55) = −2.70, *p* = .009). In addition, the mean (SD) difference in writing duration between the HFC and LFC conditions was 133.86 ms (237.94 ms) in children, and was 70.95 ms (196.76 ms) in adults. The discrepancy between the two conditions did not differ between children and adults (*t*(77) = 1.21, *p* = .229).

The writing latency results are presented in Figure 1(b). Since writing latency did not follow a normal distribution, as established with the Shapiro–Wilk test (for children, HFCs: *W* = 0.90, *p* = .026, LFCs: *W* = 0.95, *p* = .275; for adults, HFCs: *W* = 0.92, *p* < .001, LFCs: *W* = 0.92, *p* = .001), Wilcoxon signed rank tests were applied to examine differences between the two conditions. The results showed that the difference in writing latency between HFCs and LFCs was not significant in children (*Z* = −0.43, *p* = .670) or adults (*Z* = −0.33, *p* = .738).

### 5.2 NBS analysis results

**FIGURE 2:**
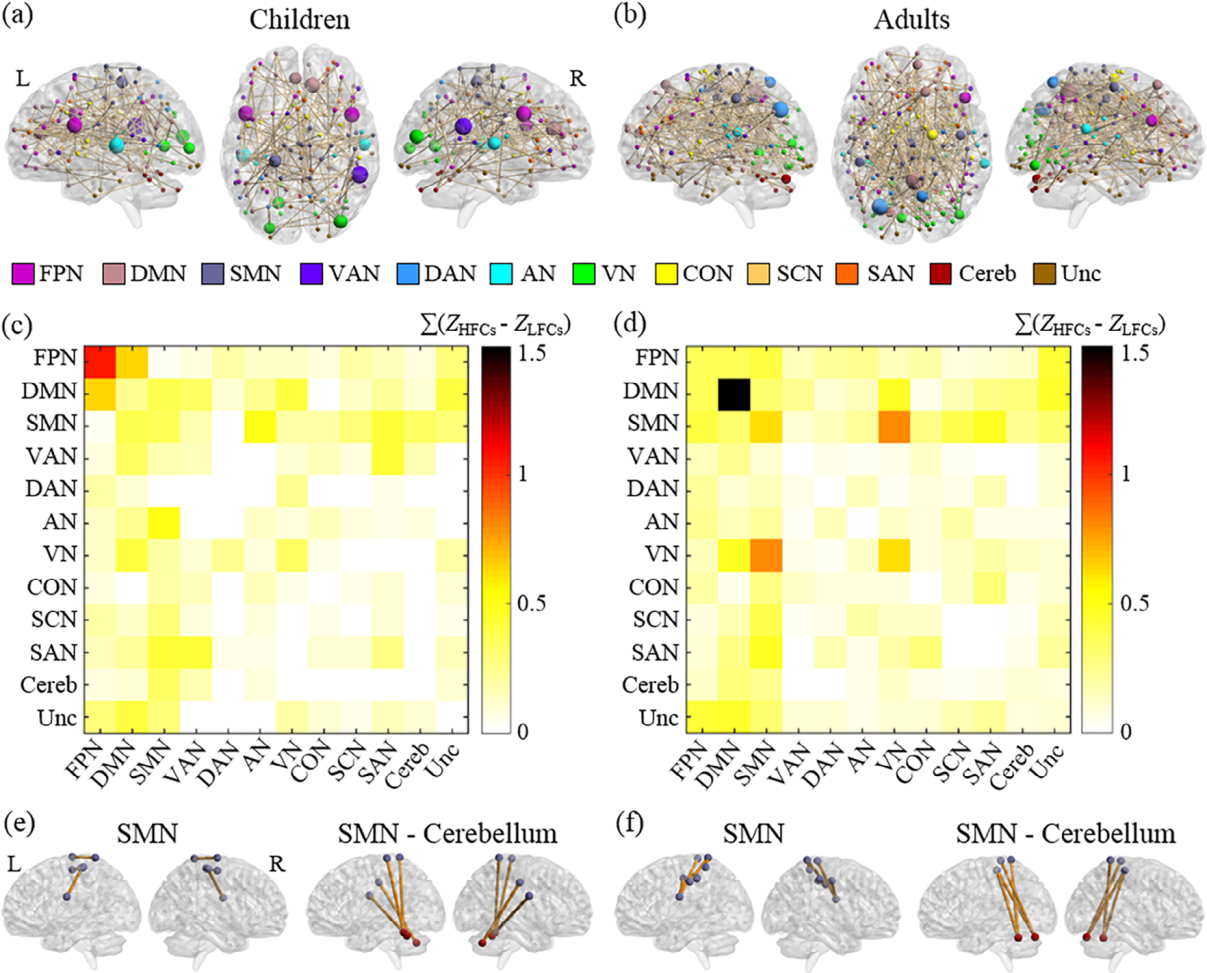
Functional brain networks that differ between the conditions of copying HFCs and LFCs. The brain plots show the functional brain network with greater connectivity in the HFC condition than the LFC condition in children (a) and adults (b). The colors of the nodes in the brain plots indicate the network to which they belong. The large nodes represent hubs, whose sizes are proportional to the node strengths. The matrix plots represent connectivity strength between pairs of the 12 brain networks in children (c) and adults (d). The color of each element in the matrices represents the sum of the weight of all the edges for the connected networks. The patterns of intranetwork connectivity within the SMN and internetwork connectivity between the SMN and cerebellar network in children (e) and adults (f) are illustrated. AN, auditory network; Cereb, cerebellar network; CON, cingulo‐opercular network; DAN, dorsal attention network; DMN, default mode network; FPN, frontal–parietal network; HFCs, high‐frequency characters; L, left; LFCs, low‐frequency characters; R, right; SAN, salience network; SCN, subcortical network; SMN, somatomotor network; Unc, uncertain; VAN, ventral attention network; VN, visual network; Z, Fisher's z scores

In children, the NBS analysis revealed that copying HFCs induced stronger network connectivity than copying LFCs in a large‐scale functional network involving 128 nodes and 144 edges, mainly encompassing intranetwork connectivity within the frontal–parietal network (FPN) and SMN, as well as internetwork connectivity between the FPN and default mode network (DMN), between the SMN and auditory network (AN), and between the SMN and cerebellar network (Figure 2(c)). Several nodes were identified as hubs, including a node in the SMN (the left postcentral gyrus), two nodes in the FPN (the left inferior frontal gyrus and right precentral gyrus), three nodes in the VN (the bilateral middle occipital gyrus and left lingual gyrus), and two nodes in the AN (the bilateral superior temporal gyrus) (Figure 2(a), see Supplementary Table S1 for the detailed information on all hubs). In particular, character frequency influenced intranetwork connectivity within the SMN and internetwork connectivity between the SMN and cerebellar network (Figure 2(e)). However, the LFC > HFC contrast failed to detect significant differences in network connectivity between the conditions.

In adults, we found that copying HFCs induced increased FC relative to copying LFCs in a large‐scale functional brain network (196 nodes and 254 edges), primarily encompassing intranetwork connectivity within the DMN, VN, and SMN, as well as internetwork connectivity between the SMN and VN and between the SMN and cerebellar network (Figure 2(d)). Several regions were identified as hubs, including three nodes in the SMN (the right precentral gyrus, left inferior parietal gyrus, and paracentral lobule), a node in the cerebellar network (the left cerebellar declive), three nodes in the VN (one in the right fusiform gyrus and two in the left lingual gyrus), and two nodes in the AN (the bilateral superior temporal gyrus) (Figure 2(b), see Supplementary Table S1 for detailed information on all hubs). In line with child findings, we also observed an effect of character frequency on intranetwork connectivity within the SMN and internetwork connectivity between the SMN and cerebellar network (Figure 2(f)).

### 5.3 Brain–behavior correlation results

In children, the regression analysis revealed that handwriting motor speed (*t*(14) = 4.48, *p* = .001), VMI quality (*t*(14) = −3.91, *p* = .002), phonological awareness (*t*(14) = −3.11, *p* = .008), orthographic awareness (accuracy for noncharacters: *t*(14) = 1.95, *p* = .072); reaction time for pseudocharacters: *t*(14) = 1.90, *p* = .078; reaction time for noncharacters: *t*(14) = −2.96, *p* = .010 and age (*t*(14) = −5.72, *p* < .001) were significantly correlated with connectivity strength between the SMN and cerebellar network (*F*(7,14) = 9.10, *p* < .001). Orthographic awareness (reaction time for pseudocharacters: *t*(18) = 2.45, *p* = .025; reaction time for noncharacters: *t*(18) = −2.76, *p* = .013) and age (*t*(18) = −1.93 *p* = .069) were significantly correlated with connectivity strength within the SMN (*F*(3,18) = 3.21, *p* = .048). Moreover, writing quality (*t*(16) = 2.04, *p* = .059), reading fluency (*t*(16) = −1.83, *p* = .085), orthographic awareness (reaction time for noncharacters: *t*(16) = −3.65, *p* = .002), sex (*t*(16) = −3.71, *p* = .002) and age (*t*(16) = −3.01, *p* = .008) were significantly correlated with connectivity strength between the SMN and AN (*F*(5,16) = 4.56, *p* = .009).

In adults, the regression analysis showed that VMI quality (*t*(54) = −2.42, *p* = .019) was associated with connectivity strength within the SMN (*F*(1,54) = 5.86, *p* = .019). In addition, orthographic awareness (accuracy for noncharacters: *t*(49) = 2.90, *p* = .006; reaction time for pseudocharacters: *t*(49) = −3.05, *p* = .004) was associated with connectivity strength between the SMN and VN (*F*(2,49) = 6.20, *p* = .004).

### 5.4 Validation results

The results of validation procedures indicated that the main findings in the functional brain networks related to the interaction between central and peripheral processes were largely replicated by alternative analysis procedures in both child and adult participants. We also found that the connectivity within and between the SMN, cerebellar network, DMN, VN, and AN was modulated by character frequency, although the number of the connectivity edges were reduced under the stringent threshold of edge definition. These findings confirmed the robustness of our main findings (see Supplementary Figures S1 and S2).

Regarding the effects of head motion, the paired sample *t* tests revealed no significant differences in mean FD between the HFC and LFC conditions either in children (*t*(22) = −0.11, *p* = .911) or adults (*t*(55) = −0.50, *p* = .620). Additionally, the regression analysis found no significant correlations between the mean FD of the whole task run and the connectivity strength of the functional brain networks in relation to the effect of frequency in children (*t*(19) = −0.37, *p* = .713) or adults (*t*(52) = −0.29, *p* = .771) after controlling for age and sex. Finally, we found that these findings were replicated when the mean FD was included as a covariate (Supplementary Figure S3).

## 6 DISCUSSION

This study examined the functional brain networks underlying the interaction between central and peripheral processes involved in Chinese handwriting in children and adults. We found that the manipulation of character frequency (a central variable) elicited reconfiguration of the brain networks for motor processes, including the SMN and cerebellar network, in both children and adults. Moreover, the connectivity strength of these motor‐related networks was correlated with both motor and linguistic factors, suggesting that the central and peripheral interaction involved in handwriting was implemented in a distributed brain network (Kandel et al., 2013; Kandel & Perret, 2015; Planton et al., 2013; Zhang & Feng, 2017). Finally, we found that children and adults exhibited qualitative differences in FC between motor and cognitive‐linguistic brain networks, implying that the brain systems for the interaction between central and peripheral processes involved in handwriting vary with age. Together, these findings illuminate the brain interface supporting the interaction between central and peripheral processes in a logographic language for the first time, advancing our understanding of the neural architecture of handwriting.

Consistent with previous studies on children (Kandel & Perret, 2015) and adults (Delattre et al., 2006; Kandel et al., 2013; Kandel & Perret, 2015; Palmis et al., 2019), in‐scanner behavioral results showed that the writing duration of LFCs was higher than that of HFCs in both children and adults. Writing duration is considered to be an index of motor processing (Delattre et al., 2006; Kandel & Perret, 2015), suggesting that manipulation of the central variable results in cascading influence in motor processing. Moreover, this interaction effect was present at age 10 in Chinese children, which coincides with findings in French children (Kandel & Perret, 2015).

However, we did not detect an effect of frequency on writing latency. This result seems inconsistent with previous studies of Chinese handwriting showing that the writing latency of LFCs is higher than that of HFCs (Zhang & Wang, 2014). One plausible explanation is the differences in task paradigms. The copying task used in this study is less complex than the written naming task used by Zhang and Wang (2014) because copying does not require lexical and orthographic selection. Thus, this measurement of writing latency may not be sensitive enough to detect the effect of frequency in the copying task. Alternatively, we found that writing latency was not normally distributed in either children or adults. Thus, another possibility is that the high intersubject variance in writing latency reduced the statistical power for detecting an effect of frequency on writing latency.

At the neural level, we identified a functional brain network related to the effect of character frequency on Chinese handwriting in children and adults. Crucially, we found that the frequency triggered functional shifts in motor brain networks, providing neural evidence of the interaction between central and peripheral processes involved in Chinese handwriting. Unexpectedly, this effect of frequency manifested as increased FC exhibited by the high‐frequency condition compared to that of the low‐frequency condition. This result parallels previous findings indicating that the efficiency of functional integration is positively correlated with cognitive performance, as writing speed was faster in the high‐frequency condition than in the low‐frequency condition (Shine et al., 2016). However, this result seems to contradict the findings derived from regional activation involved in Chinese handwriting (Yang et al., 2018) and reading (Kuo et al., 2004); these studies reported that the processing of LFCs elicits greater brain responses than that of HFCs. One possible explanation for the discrepancy is the difference in data analysis approaches. In this study, we used a NBS analysis that fully captured synchronization across multiple and overlapping functional networks. The univariate brain activation analysis used by Yang et al. (2018) and Kuo et al. (2004) reflected differences in local brain activity related to the frequency of characters. It is possible that brain activation for LFCs in particular regions is stronger than that for HFCs, whereas the synchronization of activity across brain regions is stronger for HFCs than for LHCs. Following this reasoning, we infer that the increased FC for HFCs may reflect the intrinsic characteristics of their neural representations, established by learning experience or frequent usage, since no explicit lexical processing is required in the copying task. Along these lines, previous studies have shown that visual experience with words can enhance functional integration between multiple brain networks (Lopez‐Barroso et al., 2020). However, further studies are required to test this hypothesis.

We found that the connectivity within the SMN and the connectivity between the SMN and cerebellar network were commonly involved with the interaction in both children and adults. This result is consistent with an fMRI study showing that 8‐ to 11‐year‐old children as well as adults recruit a similar brain network for handwriting, despite differences in the strength of regional activation strength (Palmis et al., 2021). The shared neural basis for the interaction of central and peripheral processes in children and adults may result from the maturation of motor skills in children. This idea is consistent with previous findings showing that graphomotor skills become fast and automatic at approximately 10–11 years old (Halsband & Lange, 2006).

First, the connectivity within the SMN was modulated by character frequency; this network is correlated with both linguistic and motor skills, suggesting that it is the neural substrate for the interaction between central and peripheral processes involved in handwriting. Several regions within the SMN were identified as hubs, including the left postcentral gyrus, left inferior parietal lobule, right precentral gyrus, and left paracentral lobule; these brain regions are involved in the planning, execution and monitoring of the motor aspects of handwriting (Brownsett & Wise, 2010). Due to the high complexity of Chinese characters, the right precentral gyrus may be additionally recruited to support the processing demands of motor control. In line with this idea, previous studies have shown that the activation of the precentral gyrus ipsilateral to the writing hand supports the increased demand for motor precision (Buetefisch et al., 2014; Verstynen et al., 2005). Moreover, the paracentral lobule, located within the left postcentral gyrus, plays an important role in somatosensory feedback, a necessary process for guiding motor control in handwriting (Sakurai et al., 2007; Yang et al., 2021).

In addition, we found that the coupling between the SMN and the left cerebellum was also modulated by character frequency. Correlation analysis further indicated that the connectivity between the SMN and cerebellar network was correlated with motor speed and linguistic skills in children. The engagement of the left cerebellum in Chinese handwriting has been repeatedly evidenced and is thought to be related to the high level of motor complexity (Li et al., 2021; Yang et al., 2019). The cerebellum is a well‐known motor region that supports the integration of information from motor and sensory cortices and coordinates motor output (Daskalakis et al., 2004; Ohyama et al., 2003). Furthermore, the connectivity between the left cerebellum and right parietal cortex supports the online integration of visual and kinesthetic information during human motor perception (Hagura et al., 2009). Thus, the internetwork connectivity between the SMN and cerebellar network is another neural platform that supports the interaction between central and peripheral processes involved in Chinese handwriting.

Notably, there may be an alternative explanation for our results. HFCs are also likely to be more frequently written than LFCs, and thus the frequency effect may also reflect differences in motor familiarity or complexity, that is, the size of the motor chunk. In other words, the connectivity between the SMN and cerebellar network may just reflect differences in motor processing between the HFC and LFC conditions, rather than a cascade of lexical information to motor execution. However, the results and experimental design of the present study and the characteristics of the Chinese writing system rule out this possibility. First, we found that the functional brain network related to the frequency effect included brain nodes for both sensorimotor processing (the precentral and postcentral gyrus) and linguistic processes (the superior temporal gyrus and inferior frontal gyrus). Furthermore, the connectivity of the motor networks was correlated with phonological or orthographic factors. These findings suggest that the brain networks related to the frequency effect are not solely derived from the motor discrepancy. Next, multiple studies have suggested that the motor unit of Chinese handwriting in both children (Lau, 2019) and adults (Han et al., 2007) is the radical or logographeme, both of which are sublexical orthographic units. Considering the processing efficiency of the handwriting system, it is reasonable that sublexical‐sized units serve as motor units because only a limited number of small units must be stored and later assembled to produce a large number of words (Afonso et al., 2018). Therefore, it is unlikely that all characters used in this study exist as motor chunks for handwriting. Consistent with this view, a prior behavioral study of Chinese handwriting showed that the cascade of lexicality into motor processing occurred at the radical level (the first radical) (Zhang & Feng, 2017). Finally, we asked the participants to write in a stroke‐by‐stroke fashion, which constrained the use of stroke as motor chunks in the present copying task. The number of strokes in the HFC and LFC conditions was matched. Thus, we conclude that the observed differences in the functional brain network are unlikely to be solely derived from differences in motor familiarity.

Finally, we also observed differences in functional networks underlying the frequency effect between children and adults. First, we found that the connectivity between the SMN and AN (centering on the bilateral superior temporal gyrus) was more salient in children than in adults. The superior temporal gyrus is a language region that plays a vital role in phonological processing (Arrington et al., 2019; Feng et al., 2020). Phonological representations of HFCs are more likely to be activated than those of LFCs. Thus, the connectivity between the SMN and AN may reflect the cascade of automatically activated phonological information into motor execution involved in handwriting (Afonso & Alvarez, 2011; Qu et al., 2011). Consistent with this idea, previous studies on Chinese reading have demonstrated that the connectivity of the temporal regions is stronger in children than in adults (Liu et al., 2018), suggesting that children rely more on phonological processing than adults. Moreover, unlike adults, children exhibited strong intranetwork connectivity within the FPN. The FPN is a core brain network for cognitive control (Dosenbach et al., 2007) and dynamically interacts with other networks adapted to processing demands (Cole et al., 2013). The development of the FPN extends into later adolescence (Velanova et al., 2008), manifesting as increasingly segregated connectivity (Gu et al., 2015). Due to underdeveloped executive control, children may need to exert more cognitive effort to deal with the differences in lexical processing between HFCs and LFCs, leading to increased FC within the FPN.

In contrast, connectivity between the SMN and VN (centering on the left lingual gyrus) was particularly strong in adults. The lingual gyrus is a part of the dorsal route for visuospatial processing involved in the recognition of Chinese characters (Cao et al., 2013; Sun et al., 2011). Previous studies have shown that the connectivity of occipital regions is stronger in adults than in children, implying an adult preference for visual processing while reading in Chinese (Liu et al., 2018). From this perspective, the connectivity between the SMN and VN for the frequency effect observed in adults reflects the cascade of orthographic representation to the motor process involved in handwriting. This notion is supported by the finding that the frequency effect involved in Chinese handwriting occurs at the orthographic level in adults (Qu et al., 2016). In addition, we found that the connectivity within the DMN predominantly exhibited the effect of character frequency in adults. The connectivity within the DMN increases with age (Fair et al., 2008; Fan et al., 2021), and its development facilitates improvements in cognitive function, including autobiographical memory (Mevel et al., 2013), language (Yang et al., 2013) and working memory (Sambataro et al., 2010). In particular, the DMN underpins general memory processing (Shapira‐Lichter et al., 2013). The accessibility of semantic information is higher for HFCs than LFCs. Thus, the involvement of the DMN in the frequency effect may arise from the level of semantic memory (Almeida et al., 2007; Bonin & Fayol, 2002). Previous studies have shown that adults exhibit greater FC between visual‐related and semantic‐related brain regions and decreased connectivity in phonological information networks when reading in Chinese (Zhou et al., 2021). Therefore, the link between the frequency effect and connectivity within the DMN in adults is not surprising because the semantic activation of characters is more efficient and sufficient in adults than in children, which leads to the high possibility that semantic information cascades into motor processes involved in handwriting.

However, caution should be taken when interpreting the differences in functional brain networks between children and adults as neural development. For one thing, such group differences were not derived from direct statistical comparisons between groups due to the differences in experimental design, and thus, several possible confounds are required to be taken into account. For example, handwriting task may be more challenging in children than in adults, and thus, the observed differences in the FPN and DMN between children and adults may reflect the general task difficulty. However, although children showed longer writing duration than adults in both the HFCs and LFCs conditions during fMRI, the differences in writing duration between the two conditions did not differ between age groups. Therefore, it is unlikely that functional brain networks related to the effect of character frequency were solely originated from the differences in task difficulty.

## 7 LIMITATIONS

The present study has several limitations. First, it should be noted that due to the differences in the design of the copying task, no direct statistical comparisons between the two age groups could be conducted. Consequently, the observed differences in network connectivity between children and adults are qualitative, and thus only possess implications for the development of the interaction between central and peripheral processes. A second problem is that the sample size was not matched between the two groups of participants. The differences in sample size between children and adults may bias the statistical power to detect functional brain networks related to the frequency effect. To further elucidate the full course of the neural development that underlies the interaction between central and peripheral components, future longitudinal studies with an adequate and matched sample size are needed. In particular, it would be necessary to include younger participants who have just started to learn handwriting.

Additionally, the present study only examined the interaction between central and peripheral processes involved in handwriting by manipulating a lexical‐level variable. Thus, it remains unclear whether and by what mechanisms sublexical variables influence motor execution. Prior studies in alphabetic languages have demonstrated that lexical and sublexical variables affect motor execution involved in handwriting in different manners (Kandel & Perret, 2015; Planton et al., 2013). Thus, it would be intriguing to examine how linguistic variables have a cascading influence on motor processing at the subcharacter level as well as the development of this influence with age.

Finally, the sluggishness of the BOLD response to neural activity and the low temporal resolution of the fMRI protocol placed limits on the study outcomes. In contrast to noninvasive electrophysiological recording methods such as electroencephalography (EEG) and magnetoencephalography (MEG), the present fMRI results were insufficient to temporally disentangle the neural activity associated with central and peripheral processes. New high‐temporal resolution fMRI methods are under active development (Hsu et al., 2017); these methods, together with the use of EEG and MEG, can hopefully shed further light on this question.

## 8 CONCLUSION

This fMRI‐based study identified novel neural evidence for an interaction between central and peripheral processes involved in Chinese handwriting. The connectivity within the SMN and the connectivity between the SMN and cerebellar network serve as shared brain mechanisms of this interaction in children and adults, suggesting that the interaction is found in both developing and skilled writers. This study revealed that the interaction between central and peripheral processes in Chinese handwriting (a logographic language) relies on a large‐scale reconfiguration of functional brain networks, deepening our understanding of the neural architecture of handwriting.

## AUTHOR CONTRIBUTIONS

Junjun Li: Conceptualization, methodology, visualization, validation, formal analysis, investigation, writing‐original draft. Ying Liu: Methodology, resources, investigation, formal analysis, writing‐original draft. Yi Wang: Conceptualization, methodology, writing‐review and editing. Nizhuan Wang: Conceptualization, methodology, funding acquisition. Yuzhu Ji: Conceptualization, formal analysis, methodology. Tongqi Wei: Conceptualization, project administration, writing‐review and editing, funding acquisition. Hong‐Yan Bi: Conceptualization, writing‐review and editing, Funding acquisition. Yang Yang: Conceptualization, methodology, funding acquisition, resources, writing‐review and editing, supervision.

## CONFLICT OF INTEREST

The authors declare that they have no known competing financial interests or personal relationships that could have appeared to influence the work reported in this article.

## Supporting information


**Figure S1** Functional brain networks that differ between the conditions of copying high‐ and low‐frequency characters, obtained by replacing the primary threshold with *p* < .005. The brain plots show functional networks with greater connectivity in the HFC condition than the LFC condition in children (a) and adults (b). The colors of the nodes in the brain plots indicate the network to which they belong. The large nodes represent hubs, whose sizes are proportional to the node strengths. The matrix plots represent connectivity strength between pairs of the 12 brain networks in children (c) and adults (d). The color of each element in the matrices represents the sum of the weight of all edges for the connected networks. HFCs, high‐frequency characters; L, left; LFCs, low‐frequency characters, R, right; *Z,* Fisher's z scores.

**Figure S2** Functional brain networks that differ between the conditions of copying high‐ and low‐frequency characters, obtained by replacing the estimation method with NBS extent. The brain plots show the functional networks greater connectivity in the HFC condition than the LFC condition in children (a) and adults (b). The colors of the nodes in the brain plots indicate the network to which they belong. The large nodes represent hubs, whose sizes are proportional to the node strengths. The matrix plots represent connectivity strength between pairs of the 12 brain networks in children (c) and adults (d). The color of each element in the matrices represents the sum of the weight of all edges for the connected networks. HFCs, high‐frequency characters; L, left; LFCs, low‐frequency characters, R, right; *Z,* Fisher's z scores.

**Figure S3** Functional brain networks that differ between the conditions of copying high‐ and low‐frequency characters obtained by including mean FD as a covariate. The brain plots show the functional brain networks greater connectivity in the HFC condition than the LFC condition in children (a) and adults (b). The colors of the nodes in the brain plots indicate the network to which they belong. The large nodes represent hubs, whose sizes are proportional to the node strengths. The matrix plots represent connectivity strength between pairs of the 12 brain networks in children (c) and adults (d). The color of each element in the matrices represents the sum of the weight of all edges for the connected networks. HFCs, high‐frequency characters; L, left; LFCs, low‐frequency characters, R, right; *Z,* Fisher's z scores.

**Table S1** Hubs in the functional brain networks associated with the effect of character frequency

## Data Availability

The data that support the findings of this study are available from the corresponding author upon reasonable request.
