# Supplementary figures and images for: Functional brain networks underlying the interaction between central and peripheral processes involved in Chinese handwriting in children and adults
Source: Hum Brain Mapp. 2022 Aug 25;44(1):142–55. doi: 10.1002/hbm.26055 (PMC9783426; doi:10.1002/hbm.26055)

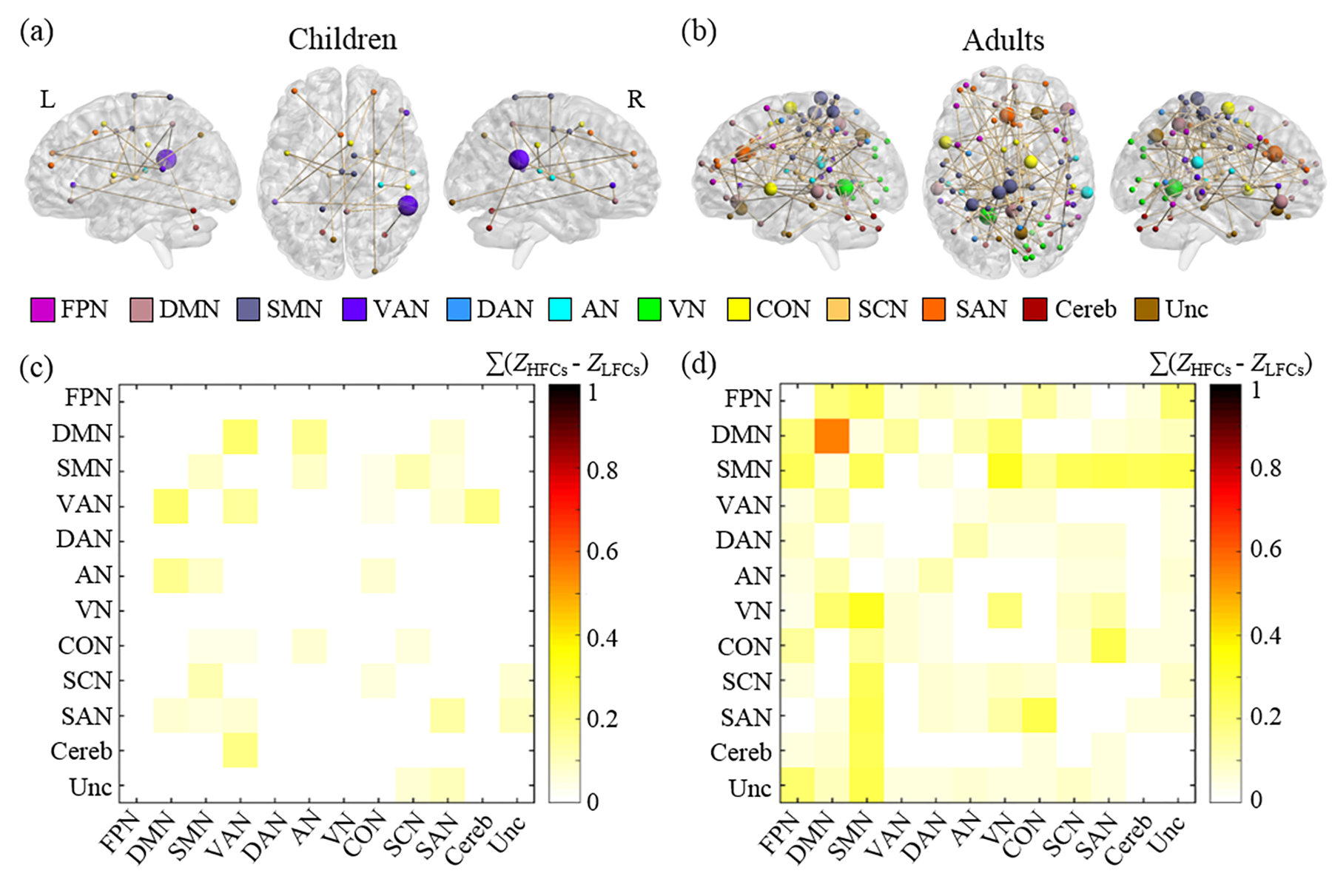

Supplement: Supplementary file 1 — Figure S1 Functional brain networks that differ between the conditions of copying high‐ and low‐frequency characters, obtained by replacing the primary threshold with p < .005. The brain plots show functional networks with greater connectivity in the HFC condition than the LFC condition in children (a) and adults (b). The colors of the nodes in the brain plots indicate the network to which they belong. The large nodes represent hubs, whose sizes are proportional to the node strengths. The matrix plots represent connectivity strength between pairs of the 12 brain networks in children (c) and adults (d). The color of each element in the matrices represents the sum of the weight of all edges for the connected networks. HFCs, high‐frequency characters; L, left; LFCs, low‐frequency characters, R, right; Z, Fisher's z scores. [file HBM-44-142-s004.tif]

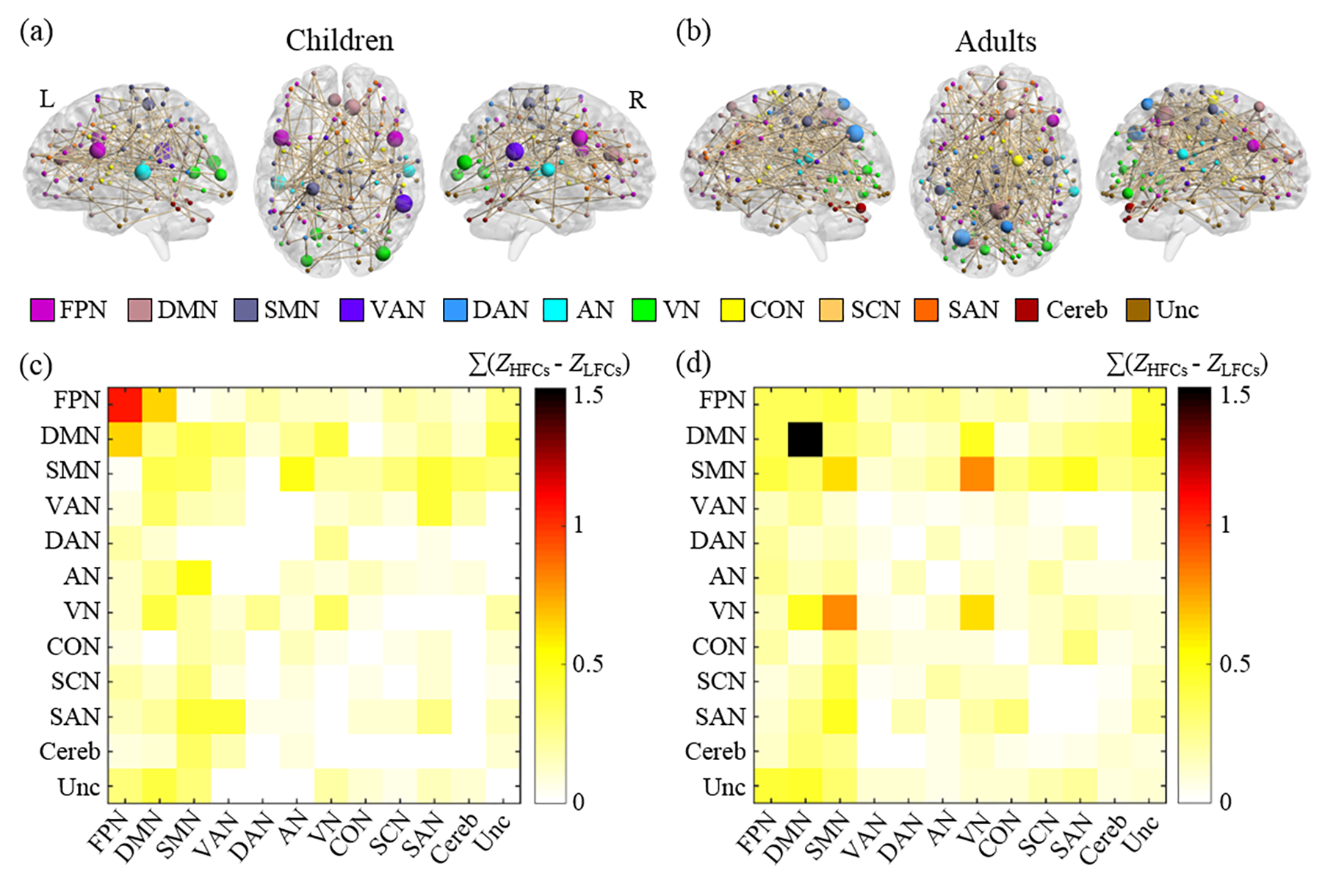

Supplement: Supplementary file 2 — Figure S2 Functional brain networks that differ between the conditions of copying high‐ and low‐frequency characters, obtained by replacing the estimation method with NBS extent. The brain plots show the functional networks greater connectivity in the HFC condition than the LFC condition in children (a) and adults (b). The colors of the nodes in the brain plots indicate the network to which they belong. The large nodes represent hubs, whose sizes are proportional to the node strengths. The matrix plots represent connectivity strength between pairs of the 12 brain networks in children (c) and adults (d). The color of each element in the matrices represents the sum of the weight of all edges for the connected networks. HFCs, high‐frequency characters; L, left; LFCs, low‐frequency characters, R, right; Z, Fisher's z scores. [file HBM-44-142-s002.tif]

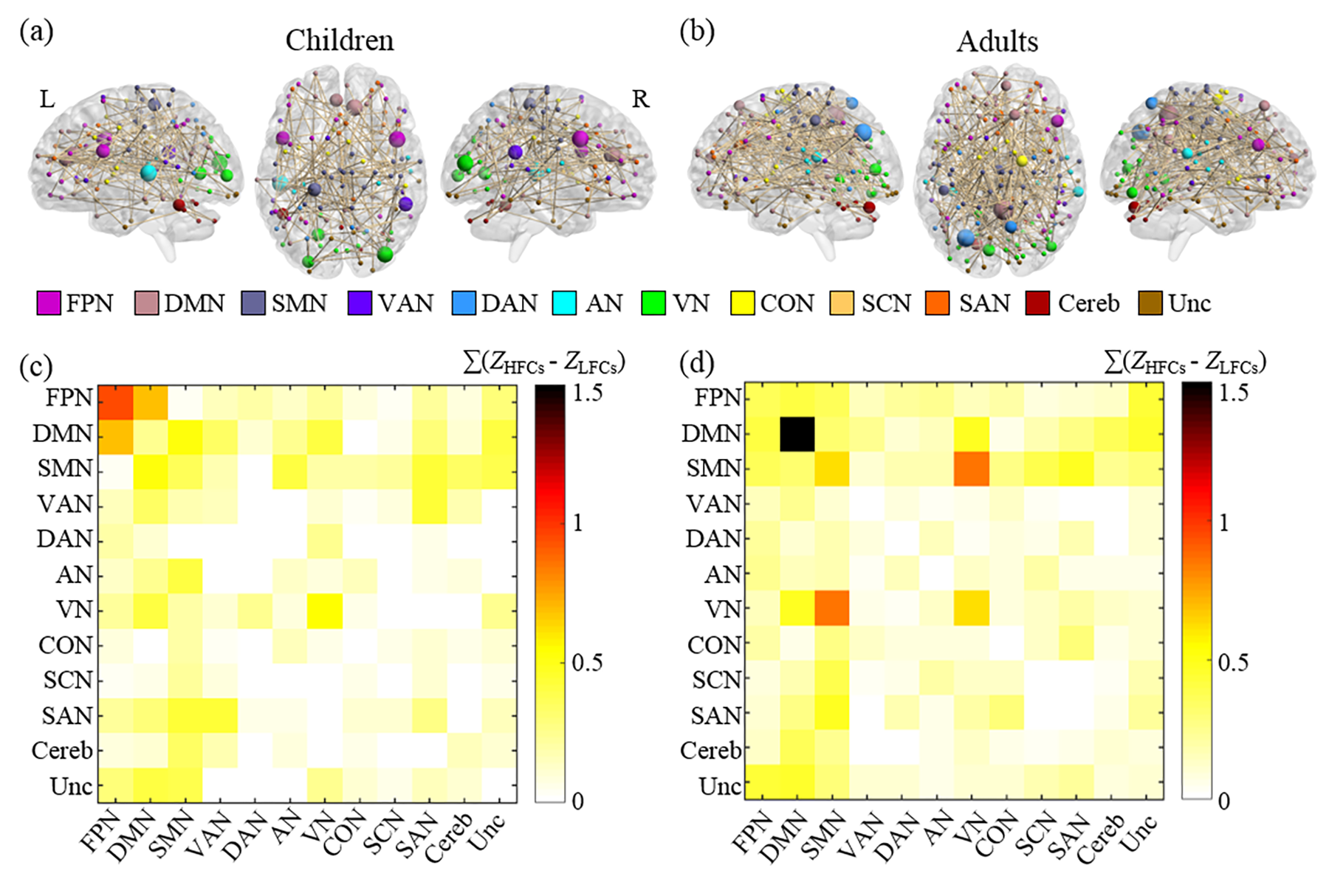

Supplement: Supplementary file 3 — Figure S3 Functional brain networks that differ between the conditions of copying high‐ and low‐frequency characters obtained by including mean FD as a covariate. The brain plots show the functional brain networks greater connectivity in the HFC condition than the LFC condition in children (a) and adults (b). The colors of the nodes in the brain plots indicate the network to which they belong. The large nodes represent hubs, whose sizes are proportional to the node strengths. The matrix plots represent connectivity strength between pairs of the 12 brain networks in children (c) and adults (d). The color of each element in the matrices represents the sum of the weight of all edges for the connected networks. HFCs, high‐frequency characters; L, left; LFCs, low‐frequency characters, R, right; Z, Fisher's z scores. [file HBM-44-142-s003.tif]
