# Supplementary material for: Functional brain networks underlying the interaction between central and peripheral processes involved in Chinese handwriting in children and adults
Source: Hum Brain Mapp. 2022 Aug 25;44(1):142–55. doi: 10.1002/hbm.26055 (PMC9783426; doi:10.1002/hbm.26055)
Supplement: Supplementary file 4 — Table S1 Hubs in the functional brain networks associated with the effect of character frequency [file HBM-44-142-s001.docx]

**Table S1** Hubs in the functional brain networks associated with the effect of character frequency

| Brain region | Coordinates in Talairach space | | | Node strength | Network |
| --- | --- | --- | --- | --- | --- |
|  | X | Y | Z |  |  |
| **Children: HFCs > LFCs** |  |  |  |  |  |
| Superior temporal gyrus | 51 | -45 | 22 | 0.51 | VAN |
| Anterior cingulate | 11 | 30 | 24 | 0.46 | DMN |
| Superior temporal gyrus | -47 | -28 | 5 | 0.46 | AN |
| Precentral gyrus | 44 | 5 | 35 | 0.46 | FPN |
| Inferior frontal gyrus | -45 | 7 | 24 | 0.45 | FPN |
| Middle occipital gyrus | 35 | -84 | 11 | 0.42 | VN |
| Superior temporal gyrus | 55 | -19 | 10 | 0.39 | AN |
| Postcentral gyrus | -21 | -34 | 58 | 0.37 | SMN |
| Lingual gyrus | -17 | -68 | 3 | 0.37 | VN |
| Anterior cingulate | -3 | 36 | 20 | 0.36 | DMN |
| Middle occipital gyrus | -25 | -89 | 0 | 0.34 | VN |
| **Adults: HFCs > LFCs** |  |  |  |  |  |
| Precuneus | 3 | -50 | 48 | 0.56 | DMN |
| Sub-gyral | -26 | -71 | 33 | 0.49 | DAN |
| Middle frontal gyrus | 45 | 19 | 30 | 0.35 | FPN |
| Superior parietal lobule | 8 | -63 | 57 | 0.33 | DAN |
| Middle frontal gyrus | 17 | -12 | 63 | 0.31 | CON |
| Cingulate gyrus | 7 | -50 | 29 | 0.31 | DMN |
| Fusiform gyrus | 41 | -78 | -12 | 0.30 | VN |
| Lingual gyrus | -15 | -53 | -2 | 0.30 | VN |
| Superior frontal gyrus | 11 | 24 | 60 | 0.30 | DMN |
| Superior temporal gyrus | 62 | -36 | 21 | 0.30 | AN |
| Inferior parietal lobule | -44 | -34 | 44 | 0.29 | SMN |
| Lingual gyrus | -8 | -80 | 5 | 0.29 | VN |
| Superior temporal gyrus | -37 | -35 | 16 | 0.29 | AN |
| Precentral gyrus | 41 | -12 | 57 | 0.29 | SMN |
| Medial frontal gyrus | 5 | 48 | 21 | 0.29 | DMN |
| Cerebellum | -16 | -75 | -25 | 0.29 | Cerebellar network |
| Cingulate gyrus | -1 | 25 | 30 | 0.28 | SAN |
| Paracentral lobule | -1 | -18 | 46 | 0.27 | SMN |
